# LigMerge: A Fast Algorithm to Generate Models of Novel Potential Ligands from Sets of Known Binders

**DOI:** 10.1111/j.1747-0285.2012.01414.x

**Published:** 2012-09

**Authors:** Steffen Lindert, Jacob D Durrant, J Andrew McCammon

**Affiliations:** 1 Department of Pharmacology, University of California San DiegoLa Jolla, CA 92093, USA; 2 Department of Chemistry and Biochemistry, University of California San DiegoLa Jolla, CA 92093, USA; 3 Department of Chemistry and Biochemistry, NSF Center for Theoretical Biological Physics, National Biomedical Computation Resource, University of California San DiegoLa Jolla, CA 92093, USA; 4 Howard Hughes Medical Institute, University of California San DiegoLa Jolla, CA 92093, USA

**Keywords:** biophysical chemistry, drug design, structure-based drug design

## Abstract

One common practice in drug discovery is to optimize known or suspected ligands in order to improve binding affinity. In performing these optimizations, it is useful to look at as many known inhibitors as possible for guidance. Medicinal chemists often seek to improve potency by altering certain chemical moieties of known/endogenous ligands while retaining those critical for binding. To our knowledge, no automated, ligand-based algorithm exists for systematically ‘swapping’ the chemical moieties of known ligands to generate novel ligands with potentially improved potency. To address this need, we have created a novel algorithm called ‘LigMerge’. LigMerge identifies the maximum (largest) common substructure of two three-dimensional ligand models, superimposes these two substructures, and then systematically mixes and matches the distinct fragments attached to the common substructure at each common atom, thereby generating multiple compound models related to the known inhibitors that can be evaluated using computer docking prior to synthesis and experimental testing. To demonstrate the utility of LigMerge, we identify compounds predicted to inhibit peroxisome proliferator–activated receptor gamma, HIV reverse transcriptase, and dihydrofolate reductase with affinities higher than those of known ligands. We hope that LigMerge will be a helpful tool for the drug design community.

Given the exponential growth of computer speed and power, the role computers play in modern drug discovery, already important, is likely to increase in prominence in coming years. Virtual screening is one application of computer-aided drug design that is already commonplace. Rather than testing millions of compounds in high-throughput screens, experiments that are costly in both time and treasure, many researchers first use docking programs to predict small-molecule binding *in silico*. Virtual screening approaches enrich a pool of candidate ligands for true binders; only a limited number of the best-scoring compounds are then tested experimentally, leading to greater hit rates and decreased cost (1–3). These methodologies have been used successfully to identify many experimentally validated ligands, including inhibitors of *Trypanosoma brucei–*RNA editing ligase 1 (4,5), *T. brucei* UDP-galactose 4′-epimerase (6), *T. brucei* farnesyl diphosphate synthase (7), *M. tuberculosis* dTDP-6-deoxy-l-lyxo-4-hexulose reductase (8), and *H. sapiens* stromelysin-1 (9).

Critical to any virtual screening project is the selection of a good database of small-molecule models whose real-world counterparts are readily available for experimental validation. These databases generally consist of compounds carefully designed to represent diverse scaffolds (i.e., diversity sets), compounds derived from common reactions (combinatorial libraries), compounds with known pharmacological properties (e.g., the set of all approved drugs), or analogs of known ligands.

In part because of the advent of high-throughput screening, many protein receptors are associated with a plethora of experimentally validated ligands (10). In designing novel small-molecule databases for virtual screening, it makes sense to consider the pharmacophoric features of known ligands. New ligands that combine the observed features of validated binders are more likely to be potent binders themselves.

BREED (11), an algorithm developed by Vertex pharmaceuticals, overlays known receptor–ligand complexes to generate novel ligands that bind with improved affinity. BREED is a receptor-based algorithm that relies on the presence of high-resolution crystal or NMR structures to overlay known ligands. To our knowledge, there is no stand-alone, ligand-based tool for recombining the three-dimensional structures of known ligands into novel potential binders.

Here, we present a program called LigMerge that provides a fast and easy way to generate molecular models derived from known inhibitors without the need for information about the receptor. We expect the program will be useful for those designing custom virtual screening, small-molecule databases when many ligands, potent or otherwise, have been identified experimentally or theoretically *via* virtual screening. LigMerge is implemented in Python and so is easily editable, customizable, and platform independent. A copy can be downloaded free of charge from http://www.nbcr.net/ligmerge/.

## Materials and Methods

### The LigMerge algorithm

**Figure 1:**
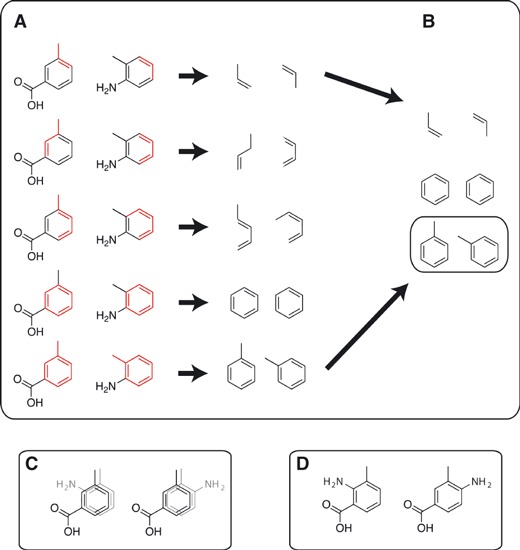
A schematic representing the LigMerge algorithm. (A) Stretches of connected atoms consisting of identical elements in sequence are identified from two distinct compounds. (B) Those stretches of connected atoms that have identical geometries are identified as common substructures. The maximum (largest) common substructure is subsequently identified (highlighted in a separate box). (C) The two distinct compounds are aligned so that their greatest common substructures are superimposed. All possible superimpositions are considered. (D) Novel compounds are generated by mixing and matching the moieties connected to each of the superimposed atoms of the maximum common substructure.

As input, LigMerge accepts two three-dimensional, PDB-formatted compound models. PDB files are the only supported input format. SDF or MOL files must be converted to the PDB format before using LigMerge. These models are processed in three steps. First, the maximum (largest) common substructure of the two models is identified (Figure 1A,B). Second, the two models are translated and rotated, so that these two substructures are superimposed (Figure 1C). Third, the two models are merged by mixing and matching the distinct fragments of each model attached at each common, superimposed atom (Figure 1D).

#### Finding the maximum common substructure (MCS)

Exhaustive lists of atom indices/element types for all heavy atoms in the two structures are first generated (Figure 1A). Hydrogen atoms are not included in this analysis. Stretches of connected atoms comprised of the same sequence of elements occurring in both structures are identified and stored, regardless of geometry. As no structural information beyond connectivity is encoded in these lists, the criterion for consideration is necessary but not sufficient for identifying a common substructure. Many of the identified common fragments will eventually be rejected for having distinct geometries, but all true common substructures are nevertheless among those enumerated. The shortest stretches considered are three-atom fragments, as shorter fragments (i.e., single atoms or mere pairs of bonded atoms) cannot reasonably be considered distinctive common substructures. Consecutively, larger fragments are likewise stored. While ideally MCSs of at least ten atoms are preferable to ensure as unique an overlay as possible, we judge three to be sufficient in extreme cases because, in addition to connectivity, the algorithm will eventually also account for the three-dimensional structures of these models. While three is set as the program default, the minimum number of common atoms can also be specified explicitly by the user.

Having identified candidate common substructures, the next step is to test for identical geometries (Figure 1B). To facilitate geometric comparison, a sorted pairwise distance matrix (i.e., a distance ‘fingerprint’) describing the distance between all atom pairs is calculated for each fragment. Two fragments are considered geometrically identical if all pairwise distances are identical within a specified tolerance. Comparisons between fragments begin with the largest candidate substructures; subsequently, smaller candidates are considered if larger candidates are found to be geometrically dissimilar. Setting the -output_mcs command-line parameter to true causes the program to output the maximum common substructure in addition to merged compound models.

Without further consideration, the above protocol ignores questions of symmetry. For example, consider two models whose greatest common substructure is a toluene. Two symmetry-related superimpositions exist (i.e., two rotations about the axis defined by the methyl-phenyl bond). The -all_symmetry_relations command-line parameter can be used to specify whether the algorithm should consider all symmetry assignments when generating merged compound models or whether it should randomly choose a single assignment from those identified (Figure 1C). The -all_symmetry_relations command-line parameter only creates multiple ligands if the overlay of the determined MCS is ambiguous.

It is important to note that LigMerge ignores ligand flexibility when performing geometric comparisons. It is therefore prudent to use ligand models of compounds in docked or crystallographic poses or to choose ligand models with inflexible segments (e.g., benzene rings). As the number of publicly available crystal structures is ever increasing and inflexible segments are common in bioactive molecules, we expect that these two limitations will not be too problematic.

#### Superimposition and fragment merging

All possible substructure assignments of maximum length and identical geometry are subsequently considered. For each, a transformation matrix is identified that minimizes the RMSD between substructures. Although only the common substructures are considered in generating this matrix, entire molecules are subjected to the transformation, essentially positioning the two models so that their maximum common substructures are superimposed (Figure 1C). When identifying the MCS, LigMerge is sensitive to the ligand conformation. If the user wishes to consider multiple ligand conformations, they need only to provide multiple pdb files representing each conformation.

Moieties from each model, comprised of fragments with atoms bound to those of the common substructure, are next identified. The common-substructure atoms to which these moieties are bound are designated ‘handle atoms’. If the command-line parameter -all_substituent_combinations is set to false, a random fragment is selected for each handle atom, and a single merged compound is generated by combining the common substructure and the selected fragments. Special consideration is given to ‘multiple-handle fragments’, that is, fragments that externally connect to two or more handle atoms. If fragments containing more than one handle atom are selected, these fragments essentially determine the selection at multiple handle–atom locations. If the command-line parameter -all_substituent_combinations is set to true, multiple merged structures with all possible combinations of fragments are generated and saved to separate PDB files (Figure 1D). If a specific fragment combination will create a molecule with steric clashes between the fragments, the merged molecule will not be generated and the fragment combination will be skipped.

### Docking of LigMerge-generated compounds

To demonstrate the utility of the LigMerge algorithm, compounds generated by applying LigMerge to a variety of known binders were docked into three receptors: peroxisome proliferator–activated receptor (PPAR) gamma, HIV reverse transcriptase (RT), and dihydrofolate reductase (DHFR).

#### Receptor preparation

Crystal structures of peroxisome proliferator–activated receptor (PPAR) gamma in complex with ligand 570 (PDB ID: 1FM9 (12)), HIV reverse transcriptase in complex with inhibitor 14 (PDB ID: 3C6T (13)), and DHFR in complex with methotrexate [PDB ID: 3DFR (14)] were used for the virtual screening studies. All crystallographic water molecules as well as the ligand molecules themselves were removed from the PDB files. Hydrogen atoms were added using PDB2PQR (15). In the case of DHFR, the hydrogen atoms associated with the NDP cofactor were derived from those present in the DUD database (10). All PQR files were then converted to the PDBQT format using MGLTools (16).

#### Ligand preparation

The BindingDB (17) was used to identify PPAR, RT, and DHFR ligands. SMILES strings of the thirty unique PPAR, RT, and DHFR ligands with the lowest IC_50_ values, respectively, were obtained from PubChem (18). The LigPrep module of Schrodinger’s Maestro computer program was used to build the molecular models in three dimensions, to add missing hydrogen atoms, and to generate all possible protonation states in a pH range of 5.0–9.0. For PPAR, LigPrep generated 30 unique molecular models from the top 15 known binders and 64 models from the top 30 binders. For RT, LigPrep generated 37 unique molecular models from the top ten known binders and 105 models from the top 30 binders. For DHFR, LigPrep generated 66 unique molecular models from the top 30 binders.

#### LigMerge compound merging

The 30 models derived from the top fifteen known PPAR inhibitors, the 37 models derived from the top ten known RT inhibitors, and the 66 models derived from the top thirty DHFR inhibitors were processed using LigMerge with the -all_symmetry_relations and -all_substituent_combinations flags set to true. The -ligands_dir flag was used to automatically run LigMerge on all possible pairs of ligands in the specified directory. Following a second LigPrep run undertaken to minimize the structures, 896, 3959, and 3974 unique potential inhibitors were identified for PPAR, RT, and DHFR, respectively.

#### Docking protocol

For PPAR, AutoDock Vina (19) was used to dock both the 896 LigMerge-generated models and the 64 models of known inhibitors into the 1FM9 binding site using a box size of 40.9 Å × 44.3 Å × 46.8 Å. For RT, the 3959 LigMerge-generated models and the 105 models of known inhibitors were docked into the 3C6T binding site using a box size of 18.0 Å × 18.0 Å × 18.0 Å. For DHFR, the 3974 LigMerge-generated compounds as well as the 66 models of known inhibitors were likewise docked into a crystallographic binding pocket (3DFR), using a box size of 42.9 Å × 44.8 Å × 44.0 Å.

#### Custom decoy libraries

For each of the LigMerge-generated ligand sets corresponding to the three receptors, the molecular weight (MW), logP, and polar surface area (PSA) were calculated using obprop (20). The set of 896 LigMerge compounds generated from known PPAR inhibitors had an average molecular weight of 611 ± 166 Da, an average logP of 7.1 ± 0.7, and an average PSA of 106 ± 38 Å^2^. The set of 3959 LigMerge compounds generated from known RT inhibitors had an average molecular weight of 493 ± 139 Da, an average logP of 4.6 ± 1.2, and an average PSA of 107 ± 40 Å^2^. Finally, the set of 3974 LigMerge compounds generated from known DHFR inhibitors had an average molecular weight of 478 ± 140 Da, an average logP of 3.6 ± 1.3, and an average PSA of 178 ± 73 Å^2^.

For each of the LigMerge-generated compound sets, an in-house script was used to generate a decoy set equal in size and chemical properties. MW, logP, and PSA statistics were calculated for each of the approximately 11 000 000 compounds of the ZINC ‘All Clean’ data set (21) using obprop (20). Subsets of the ‘All Clean’ database were then identified with chemical properties similar to those of each of the LigMerge-generated sets in terms of both averages and standard deviations. In this way, decoy libraries were generated for PPAR (896 compounds), RT (3959 compounds), and DHFR (3974 compounds) that had average MW, logP, and PSA values within 1% of the values derived for the corresponding reference LigMerge data sets of same size. PPAR was the only exception; the average molecular weight of the PPAR decoy library was within 13% (MW = 532 Da) of the reference LigMerge-generated set because there was an insufficient number of high-molecular-weight compounds in the ZINC ‘All Clean’ data set. Additionally, it was ensured that standard deviations for these quantities did not exceed values in the corresponding LigMerge-generated sets. The decoy libraries were docked into their respective receptors with the same parameters used to dock the LigMerge-generated compound sets.

## Results and Discussion

LigMerge is an open-source, easy-to-use tool for generating novel compounds with structural features similar to those of known ligands. Compounds derived from known ligands are more likely to be true binders themselves. Once generated, LigMerge-derived compounds can be docked into receptor structures to identify likely inhibitors for subsequent synthesis and experimental validation.

To demonstrate the utility of the LigMerge algorithm, three protein drug targets with many known inhibitors were chosen as test systems: peroxisome proliferator–activated receptor (PPAR) gamma, HIV reverse transcriptase (RT), and dihydrofolate reductase (DHFR). For each of these systems, novel compounds were generated using LigMerge by combining features of known inhibitors. Predicted binding affinities were then assessed by computer docking. To demonstrate that LigMerge can generate a set of compounds enriched for high-affinity binders above and beyond screens of chemically similar molecules chosen at random, we also dock appropriate decoy databases into each of the three receptors studied to facilitate comparison.

### Peroxisome proliferator–activated Receptor

**Figure 2:**
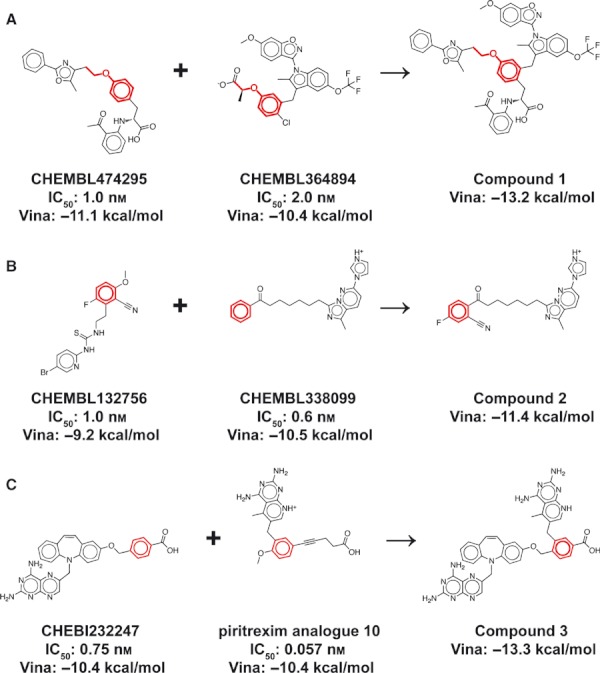
The top LigMerge-generated compounds and the known inhibitors from which they are derived. The maximum common substructures are highlighted in red. (A) Peroxisome proliferator–activated receptor gamma. (B) Reverse transcriptase. (C) Dihydrofolate reductase.

**Figure 3:**
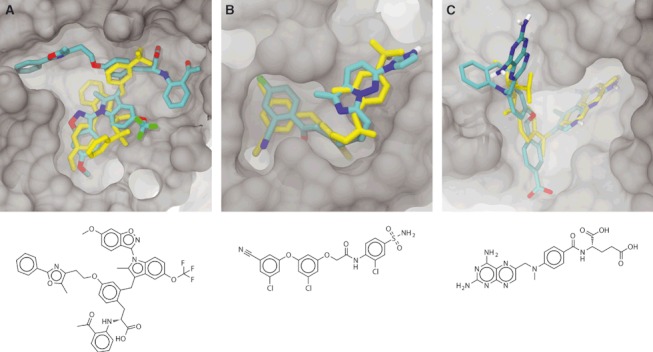
The predicted binding poses of the top LigMerge-generated compounds docked into their respective receptors. In all panels, some portions of the protein have been removed to facilitate visualization. Docked LigMerge-generated compounds are colored by element, and known co-crystallized compounds are colored yellow. The following are the standard representations of the co-crystallized ligands: (A) compound 1, docked into PPAR. The crystallographic ligand (in yellow) is compound 2a, a known binder. (B) Compound 2, docked into HIV reverse transcriptase. The crystallographic ligand (in yellow) is inhibitor 14, a known binder. (C) Compound 3, docked into dihydrofolate reductase. The crystallographic ligand (in yellow) is methotrexate, a known binder.

A total of 896 LigMerge-generated compounds were derived from the top fifteen experimentally verified PPAR binders listed in the BindingDB database (17) as of October 2011. These compounds, together with the top thirty experimentally known inhibitors, were docked into an PPAR crystal structure using AutoDock Vina (19). The best-scoring LigMerge molecule (compound **1**, Figure 2A) and known inhibitor (Figure 3A) had estimated binding affinities of −13.2 and −11.1 kcal/mol, respectively. In fact, there were 109 LigMerge-generated models that scored better than the best-known inhibitor.

The best-scoring binding pose of the top-ranked LigMerge molecule (compound **1**) is shown in Figure 3A, together with the crystallographic pose of a known inhibitor (in yellow, taken from PDB ID: 2HFP (22)). The similarities between the poses of these two compounds are noteworthy. The predicted pose of compound **1** positions benzene and anisole substructures coincident with those of the known inhibitor. Additionally, the trifluoromethyl group of compound **1** is predicted to be proximal to a sulfonamide group of the known compound, and the trifluoromethyl group of the known inhibitor is positioned proximal to the predicted location of a compound-**1** carboxylate group. Others have suggested that fluorinated methyl groups might be bioisosteres of the carboxylate group (compare PDB structures 3AEB and 3AE6) and the sulfonamide group (compare PDB structures 2XBV and 2XBX (23)). The similarities of these binding modes are not likely the result of mere chance; they give credence to the hypothesis that the LigMerge-generated compounds have improved docking scores specifically because they are based on known inhibitors and therefore build on pharmacophores known to be relevant to the receptor of interest.

**Figure 4:**
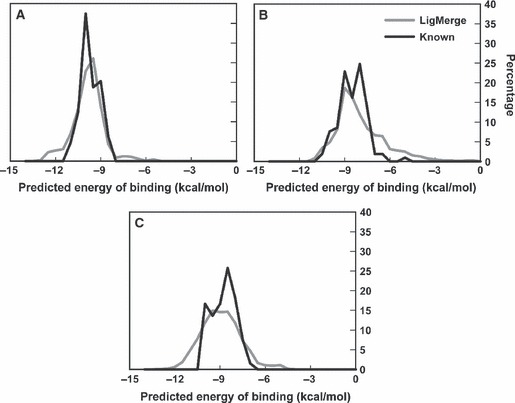
Vina score Histograms: LigMerge-generated Compounds versus Known Inhibitors. The LigMerge-generated compounds are shown in gray, and the known inhibitors are shown in black. (A) The histograms for PPAR gamma. (B) The histograms for HIV RT. (C) The histograms for DHFR.

Figure 4A shows a normalized histogram of the Vina scores associated with the LigMerge-generated and top 30/known inhibitor compounds. The docking score distribution of the LigMerge-generated compounds is markedly broader than that of the known inhibitors. As expected, LigMerge generated a number of compounds that scored worse than the known inhibitors; as compound models are generated through an exhaustive combinatorial process, it is unsurprising that some LigMerge compounds had reduced predicted binding affinities. However, the docking score distribution of the LigMerge-generated compounds also extends further toward high affinities than that of the known inhibitors. About 5% of the known inhibitors had docking scores better than −11.0 kcal/mol, suggesting tight binding. In contrast, more than 15% of the docked LigMerge compounds scored in that range, suggesting a genuine enrichment for strong binders.

### Reverse transcriptase

A total of 3959 LigMerge-generated compounds were derived from the top ten experimentally verified RT binders listed in the BindingDB database (17) as of October 2011. These compounds, together with the top thirty experimentally known inhibitors, were docked into an RT crystal structure using AutoDock Vina (19). The best-scoring LigMerge molecule (compound **2**, Figure 2B) and known inhibitor (Figure 3B) had estimated binding affinities of −11.4 and −10.5 kcal/mol, respectively. In fact, there were 132 LigMerge-generated models that scored better than the best-known inhibitor.

The best-scoring binding pose of the top-ranked LigMerge molecule (compound **2**) is shown in Figure 3B, together with the crystallographic pose of a known inhibitor (in yellow, taken from PDB ID: 3C6T (13)). The similarities between the poses of these two compounds are noteworthy. Aside from the fact that they are predicted to occupy the same general space in the binding pocket, the 3-fluorobenzonitrile moiety of compound **2** docked at the same location as the analogous 3-chlorobenzonitrile moiety of the co-crystallized inhibitor. Additionally, the aromatic imidazo[1,5-b]pyridazine moiety of compound **2** docks at the same location, and in the same plane, as a crystallographic benzene moiety of the known inhibitor. Again, the similarities of these binding modes are not likely the result of mere chance; LigMerge-generated compounds likely have improved docking scores because they are based on known inhibitors rather than chosen at random.

Figure 4B shows a normalized histogram of the Vina scores associated with the models of both the top 30 known inhibitors and the LigMerge-generated compounds. While some LigMerge-generated compounds again performed a good deal worse than known inhibitors, as expected, LigMerge did generate a number of compounds with higher-scoring predicted affinities; over 4% of the LigMerge compounds scored better than −10.0 kcal/mol, compared to fewer than 2% of the known inhibitors.

### Dihydrofolate reductase

A total of 3974 LigMerge-generated compounds were derived from the top thirty experimentally verified DHFR binders listed in the BindingDB database (17) as of October 2011. These compounds, together with the thirty experimentally known inhibitors themselves, were docked into a DHFR crystal structure. The best-scoring LigMerge molecule and known inhibitor (Figure 3C) had estimated binding affinities of −13.3 and −10.4 kcal/mol, respectively. LigMerge generated 608 models that scored better than the best-known inhibitor.

An analysis of the predicted binding pose of the top-scoring LigMerge-generated compound, compound **3** (Figure 2C), suggests that, as before, the enhanced predicted affinity over known inhibitors has not arisen by chance alone. The top predicted ligand was derived from the two best-scoring known inhibitors (Figure 2C): CHEBI232247 (24) and piritrexim analog 10 (25), with IC_50_ values of 0.75 and 0.057 nm, respectively. Additionally, the top Vina pose of compound **3** places the moiety analogous to the pteridine-2,4-diamine of methotrexate, a known inhibitor (in yellow, taken from PDB ID: 3DFR (14)), deep within the same folate-binding pocket. The novel ligand binds in ways that are similar to known inhibitors, as expected given that pharmacophoric information from known ligands has essentially been leveraged in the design of these novel compounds.

Histograms showing the Vina score distributions of the 30 known DHFR inhibitors, as well as the LigMerge-derived compound models, are shown in Figure 4C. The distribution of the Vina scores associated with the LigMerge-generated compounds was again generally wider than that of the known inhibitors. As before, some of the LigMerge compounds were certainly incompatible with potent binding, but 15.5% of the compound models were predicted to bind more potently than any known inhibitor.

#### Decoy library docking

We propose that the LigMerge-generated compound set included compounds with improved predicted affinities over those of known inhibitors because LigMerge generates novel compounds from known inhibitors in an intelligent and systematic way. However, it could be that these improved compounds were identified simply because the set of LigMerge-generated compounds was much larger than the set of known inhibitors, making it statistically more likely that a high-affinity predicted ligand would be found. To rule out this possibility, we compared the docking performance of the three LigMerge-generated compound sets to that of decoy libraries similar in size and average chemical properties.

**Figure 5:**
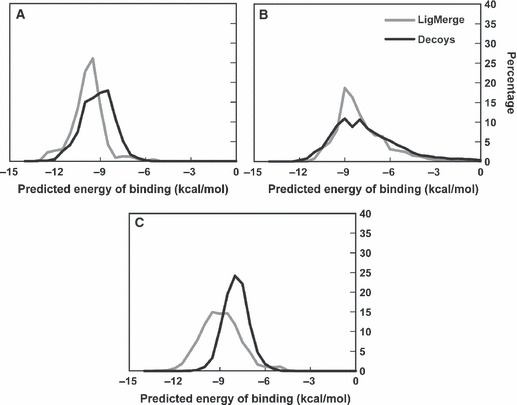
Vina score Histograms: LigMerge-generated Compounds versus Random Decoys. The LigMerge-generated compounds are shown in gray, and the decoy compounds are shown in black. (A) The histograms for PPAR gamma. (B) The histograms for HIV RT. (C) The histograms for DHFR.

Figure 5 shows normalized histograms of the Vina score distributions for LigMerge and decoy docking into PPAR (Figure 5A), RT (Figure 5B), and DHFR (Figure 5C). For PPAR and DHFR, the LigMerge distributions are clearly shifted toward higher binding affinities, suggesting legitimate enhancement beyond what would be expected by docking compounds chosen at random. In contrast, the LigMerge score distribution associated with RT is similar to that of the decoy library. This may well be a consequence of Vina’s inability to discriminate between native-like and non-native-like ligands for the HIV RT test system. These results demonstrate that for two of three systems, LigMerge provided a useful enrichment for high-affinity predicted binders.

## Conclusion

We here present an algorithm called LigMerge that considers two three-dimensional models of known or suspected small-molecule inhibitors and forms derivative models with similar chemical features. In the process of merging models, LigMerge first identifies the maximum common substructure (MCS). The MCS, which can be saved for later examination, may itself be a valuable tool for ligand evaluation. Next, the program aligns the two molecules by their mutual MCS, so that they are partially superimposed. Finally, the chemical moieties attached to each superimposed atom of the maximum common substructure are recombined, producing composite molecules similar to known or suspected inhibitors, but with potentially higher affinities. LigMerge is freely available through the National Biomedical Computation Resource (NBCR) and can be downloaded at http://www.nbcr.net/ligmerge/.
